# Resistance to empirical antibiotics in urinary tract infections caused by Enterobacterales in the East of England

**DOI:** 10.1093/jacamr/dlaf003

**Published:** 2025-01-22

**Authors:** Stuart Drazich-Taylor, Catherine Dominic, James Moore, Ashley Craine, Davis Nwaka

**Affiliations:** University of East Anglia, Research Park Norwich, Norfolk NR4 7TJ, UK; Department of Microbiology, Norfolk and Norwich University Hospital, Colney Lane, Norwich, Norfolk NR4 7UY, UK; University of East Anglia, Research Park Norwich, Norfolk NR4 7TJ, UK; Department of Microbiology, Norfolk and Norwich University Hospital, Colney Lane, Norwich, Norfolk NR4 7UY, UK; Department of Microbiology, Norfolk and Norwich University Hospital, Colney Lane, Norwich, Norfolk NR4 7UY, UK; Department of Microbiology, Norfolk and Norwich University Hospital, Colney Lane, Norwich, Norfolk NR4 7UY, UK; Department of Microbiology, Norfolk and Norwich University Hospital, Colney Lane, Norwich, Norfolk NR4 7UY, UK

## Abstract

**Objectives:**

To characterize resistance rates in urinary tract infections caused by Enterobacterales to first- and second-line antibiotics.

**Methods:**

Positive urine cultures examined by the Eastern Pathology Alliance network from September 2018 to September 2023 were retrospectively analysed. Enterobacterales from non-pregnant adults were included. Resistance to cefalexin, nitrofurantoin, trimethoprim, pivmecillinam and fosfomycin was investigated.

**Results:**

A total of 193 137 samples from 99 635 patients met inclusion criteria. The mean number of episodes per patient was 1.94, with a range of 1–55 episodes over the 5 year period. Patients were predominantly of female sex (76.6%) and of an older age (mean age 66.4 ± 19.5 SD). *Escherichia coli* was the commonest organism isolated (73%) followed by undifferentiated coliforms (16%), *Proteus* spp. (6%) and *Klebsiella pneumoniae* (2%). Across all samples, trimethoprim resistance was high at 27%, with lower cefalexin (8.3%) and nitrofurantoin (8.8%) resistance. Resistance to two or three of the first-line antibiotics—trimethoprim, cefalexin and nitrofurantoin—was 5.5% and 0.6%, respectively. In *E. coli* there was low fosfomycin resistance at 3.1%. In resistant isolates subject to extended sensitivity testing, moderate pivmecillinam (21%) resistance was demonstrated. Organisms demonstrating AmpC and ESBL resistance were detected in 3.2% and 3.5% of isolates. Trimethoprim resistance was highest at James Paget University Hospital (37%) and surrounding general practices (30%).

**Conclusions:**

This study illustrates resistance rates to commonly prescribed antibiotics for urinary tract infections in Norfolk and Waveney. Adjustments to local empirical antibiotic guidelines have been shaped by the resistance rates demonstrated herein.

## Introduction

Antimicrobial resistance (AMR) is a growing global health threat^1^ with rising resistance in Enterobacterales complicating treatment and contributing to higher morbidity and mortality.^2,3^ Monitoring resistance patterns is crucial for guiding empirical therapy and effective antimicrobial stewardship.^4^ Urinary tract infections (UTIs), caused primarily by Enterobacterales such as *Escherichia coli* and *Klebsiella pneumoniae*, are among the commonest bacterial infections worldwide.^5^ Many UTIs are managed empirically based on local resistance patterns and antibiotic guidelines, but increasing resistance leads to more frequent failures with empirical regimes.^6^ NHS England data from 2023 show that one-quarter of urine samples had bacteria resistant to a first-line antibiotic, emphasizing the need for ongoing surveillance to inform antibiotic policies.^7^

The Eastern Pathology Alliance (EPA) serves a population of 1.5 million across Norfolk and Waveney and processes microbiology samples from general practices and regional hospitals, including Norfolk and Norwich University Hospital (NNUH), Queen Elizabeth Hospital (QEH) and James Paget University Hospital (JPUH). In our region, trimethoprim and nitrofurantoin are the first-line treatments for UTIs.

To assess resistance patterns within our region, we conducted a retrospective analysis of positive urine samples over 5 years. By analysing data within our network, we sought to provide a comprehensive overview of resistance patterns and offer insights into the local epidemiology of AMR.

## Methods

Positive urine cultures examined by the EPA network from September 2018 to September 2023 were retrospectively analysed. All Gram-positive species, non-Enterobacterales Gram-negatives, yeasts and mixed cultures were excluded. All samples from children (<18 years old) and pregnant women were excluded. Samples were excluded if they were within 7 days of a previous sample for the same patient and the cultured organism was the same. Sensitivities were interrogated for cefalexin, nitrofurantoin, trimethoprim, pivmecillinam and fosfomycin.

Urine samples were processed in accordance with standard laboratory practice. All urine samples undergo fluorescence flow cytometry (Sysmex UF-5000) followed by inoculation onto orientation agar (BD BBL CHROMagar). Disc sensitivities are performed on Mueller–Hinton agar based on EUCAST guidance.^8^ The presence of AmpC and ESBL isolates was confirmed using MAST D69C, D76C and D63C detection sets.

Enterobacterales isolates showing resistance on disc testing to nitrofurantoin and trimethoprim or resistance to amoxicillin, co-amoxiclav and cefalexin or resistance to cefpodoxime undergo second-line antimicrobial testing including pivmecillinam and fosfomycin for *E. coli*. Members of the KESC group (*Klebsiella*, *Enterobacter*, *Serratia* and *Citrobacter*), which form large blue colonies on orientation agar, are identified if they meet the above resistance criteria, otherwise the culture result is reported as coliform species.

Data were analysed in R Studio Version 4.1.2. Continuous variables were reported as mean ± SD, categorical data were reported as proportions. This study was registered as a service evaluation project with the information governance team.

## Results

From September 2018 to September 2023, 193 137 samples met our inclusion criteria. These represented 99 635 patients. The mean number of episodes per patient was 1.94, with a range of 1–55 episodes over the 5͔ year period. Most patients were women (76.6%) and of an older age (mean 66.4 ± 19.5 SD). Approximately 18% (*n* = 34 523) of samples were from the three hospitals in the EPA region, with the remainder predominantly coming from general practices (82%, *n* = 157 718) as seen in Table 1.

**Table 1.** Demographics and resistance results

Characteristic	*N* = 193 137
Number of patients	99 635
Episodes per patient	Mean: 1.94; range: 1–55
Patient age, y	66.4 (mean); 19.5 (SD)
Patient sex distribution, %	
Female	76.6
Male	23.4
Nitrofurantoin, *n* (%)	
Resistant	16 098 (8.8)
Sensitive	166 964 (91)
Trimethoprim, *n* (%)	
Resistant	51 748 (27)
Sensitive	140 537 (73)
Cefalexin, *n* (%)	
Resistant	16 054 (8.3)
Sensitive	176 922 (92)
Resistance to two first-line antibiotics^a^, *n* (%)	10 615 (5.5%)
Resistance to three first-line antibiotics^a^, *n* (%)	1084 (0.6%)
Fosfomycin, *n* (%) reported in *E. coli*	
Resistant	328 (3.1)
Sensitive	10 300 (97)
Resistance to first-line antibiotics in *E. coli* isolates where fosfomycin was tested, *n* (%)	
Trimethoprim	6499 (61)
Nitrofurantoin	1677 (16)
Cefalexin	8701 (82)
Pivmecillinam, *n* (%)	
Resistant	4427 (21)
Sensitive	16 497 (79)
Resistance to first-line antibiotics in isolates where pivmecillinam was tested, *n* (%)	
Trimethoprim	12 819 (61)
Nitrofurantoin	4052 (32)
Cefalexin	12 727 (61)
AmpC present, *n* (%)	6174 (3.2)
ESBL producer, *n* (%)	6747 (3.5)
Trimethoprim resistance by specimen origin, *n* (%)	
ECCH and JPUH GP (*n* = 27 929)	8379 (30)
JPUH (*n* = 9108)	3375 (37)
NNUH (*n* = 16 660)	4658 (28)
Norwich GP and NCH&C (*n* = 87 894)	22 325 (25)
NSFT—mental health (*n* = 398)	174 (44)
Private (*n* = 498)	101 (20)
QEH (*n* = 8755)	2325 (27)
QEH GP (*n* = 41 895)	10 411 (25)

ECCH and JPUH GP, East Coast Community Healthcare and James Paget University Hospital catchment area general practice; JPUH, James Paget University Hospital; NCH&C, Norfolk Community Health and Care; NNUH, Norfolk and Norwich University Hospital; Norwich GP, Norwich catchment area general practice; NSFT, Norfolk and Suffolk Foundation Trust; QEH, Queen Elizabeth Hospital; QEH GP, Queen Elizabeth Hospital catchment area general practice.

^a^Resistance to two or more of cefalexin, nitrofurantoin or trimethoprim.

Trimethoprim resistance was high, detected in 27% (*n* = 51 748) of isolates tested. Cefalexin and nitrofurantoin resistance was lower at 8.3% (*n* = 16 054) and 8.8% (*n* = 16 098), respectively. Resistance to two or three of the first-line antibiotics—trimethoprim, cefalexin and nitrofurantoin—was 5.5% (*n* = 10 615) and 0.6% (*n* = 1084), respectively.

Pivmecillinam resistance was present in 21% (*n* = 4427) of isolates tested. Resistance to first-line antibiotics in isolates where pivmecillinam was tested was 61% for trimethoprim (*n* = 12 819), 32% for nitrofurantoin (*n* = 4052) and 61% for cefalexin (*n* = 12 727). Fosfomycin had resistance rates of 3.1% in *E. coli* (*n* = 328). Resistance to first-line antibiotics in *E. coli* isolates where fosfomycin was tested was 61% for trimethoprim (*n* = 6499), 16% for nitrofurantoin (*n* = 1677) and 82% for cefalexin (*n* = 8701). AmpC resistance mechanisms were detected in 3.2% of isolates (*n* = 6174), and 3.5% of organisms were ESBL producers (*n* = 6747).

**Figure 1.**
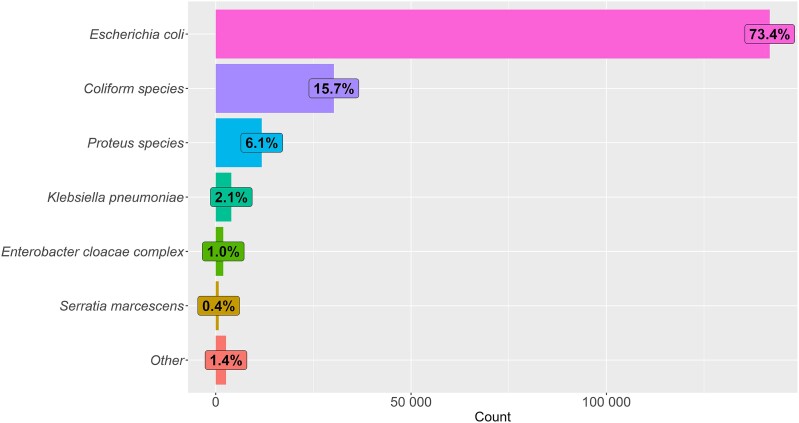
Organisms cultured from positive urine samples.

There was a variance in trimethoprim resistance across the region, with JPUH (37%, *n* = 3375) and surrounding general practices (30%, *n* = 8379) having high resistance levels. Mental health centres also demonstrated a high level of resistance, with 44% (*n* = 174) of isolates resistant to trimethoprim. The most common organism was *E. coli*, isolated in 73.4% (*n* = 141 816) of cases, followed by undifferentiated coliforms in 15.7% (*n* = 30 245) of samples. *Proteus* species were identified in 6.1% (*n* = 11 785) of cases. A smaller proportion of infections were caused by *Klebsiella pneumoniae* (2.1%, *n* *=* 3987) and *Enterobacter cloacae* complex (1%, *n* = 1925) (Figure 1).

## Discussion

Our research demonstrates the burden of resistance to empirical antibiotics in UTIs caused by Enterobacterales over the past 5 years in the East of England. Understanding these resistance patterns is essential for developing effective empirical treatment protocols.

This study highlights the challenges of diagnosing and treating UTIs. Whereas the average number of episodes per patient was 1.94 over the 5 year period, there were patients who generated many positive samples despite our efforts to minimize the impact of repeated sampling during a single episode. It is difficult to ascertain how many of these samples would have reflected a true infection in contrast to asymptomatic bacteriuria in the elderly.^9^ With the NICE no longer recommending urine dipsticks for patients aged over 65 for UTI diagnosis,^10^ there is a reliance on diagnostic gestalt when managing UTIs in the elderly. The development of novel technologies such as urinary biomarkers of infection^11^ or point-of-care tests may improve prescribing practice.^12^

Our data reveal high rates of resistance to trimethoprim at 27%, which is worse in specific regions—37% at JPUH and 44% at Norfolk and Suffolk Foundation Trust mental health units. Jancel and Dudas^13^ recommend that trimethoprim should be used as an empirical agent if resistance is below 20%. Antibiotic resistance in mental health units could be driven by a limited formulary or poor antimicrobial stewardship.^14^ JPUH is in Great Yarmouth, which is among the most deprived areas in the country, with residents having high rates of physical health conditions.^15^ This may lead to increased access of healthcare services, exposure to antibiotics and colonization with resistant flora. Resistance in the surrounding general practices may represent spillover from the hospital into the community.

In contrast to trimethoprim, nitrofurantoin and cefalexin continue to be effective agents, with resistance rates at an acceptable level of less than 10%. Given the low levels of resistance to two first-line antibiotics (5.5%), it is likely that an alternative first-line agent would be suitable even in the event of intolerance or resistance to one agent. However, cefalexin is not a recommended first-line antimicrobial in the NICE guidance for lower UTIs in males or females.^16^ Yet, cefalexin resistance rates within our cohort were low at 8.3%, and a review by Nguyen and Graber^17^ has advocated for cefalexin’s effectiveness in the treatment of lower UTIs. Our rates of AmpC and ESBL resistance were low at ∼3.5%, which is unlikely to impact on the utility of cefalexin within our empirical guidelines.

Fosfomycin resistance in *E. coli* within our dataset was low at 3.1%, which is similar to the resistance rates of 1% found in another UK centre.^18^ Pivmecillinam when tested as a second-line agent in resistant isolates demonstrated resistance rates of 21%. A French study found 10.5% pivmecillinam-resistant Enterobacterales, which may be comparable to our rates when considering different testing methodologies.^19^ Even accounting for only reporting fosfomycin and pivmecillinam sensitivities in resistant isolates, our data suggest these agents could provide an alternative to trimethoprim within empirical guidelines.

Despite its comprehensive nature, this study has several limitations. Firstly, we excluded *Pseudomonas* species and Gram-positive organisms as these are less likely to be sensitive to first-line empirical antibiotics. The relative frequency by which these cause UTIs is therefore unknown. Although we attempted to mitigate for patients with multiple positive samples, some of the included results may represent colonization rather than infection. However, these patients may have been treated by clinicians and it is important to capture the subsequent development of resistance that may have occurred. Notwithstanding these caveats, the large dataset provides a valuable reflection of the situation within our region.

### Conclusion

In conclusion, our study provides data to guide the empirical treatment of UTIs caused by Enterobacterales and highlights the need to revise empirical treatment guidelines and strengthen antimicrobial stewardship. Future efforts should focus on development of novel diagnostics to assist management of UTIs and ongoing monitoring of resistance patterns to support clinical and public health strategies.
